# BRG1 is a biomarker of hypertrophic cardiomyopathy in human heart specimens

**DOI:** 10.1038/s41598-022-11829-x

**Published:** 2022-05-17

**Authors:** Jacob C. Scherba, Marc K. Halushka, Nicholas D. Andersen, Joseph J. Maleszewski, Andrew P. Landstrom, Nenad Bursac, Carolyn Glass

**Affiliations:** 1 Department of Biomedical Engineering, Duke University, Durham, NC 27710 USA; 2 Department of Pathology, Johns Hopkins Medical Institutions, Baltimore, MD 21287 USA; 3 Division of Cardiovascular and Thoracic Surgery, Department of Surgery, Duke University Medical Center, Durham, NC 27710 USA; 4 Department of Laboratory Medicine and Pathology, Mayo Clinic, Rochester, MN 55905 USA; 5 Division of Pediatric Cardiology, Department of Pediatrics, Duke University Medical Center, Durham, NC 27710 USA; 6 Department of Pathology, Duke University Medical Center, 217AM Davison Bldg, 40 Duke Medicine Circle, Box 3712 DUHS, Durham, NC 27710 USA

**Keywords:** Mechanisms of disease, Biomarkers, Cardiac hypertrophy, Epigenetics

## Abstract

Hypertrophic cardiomyopathy (HCM) is a genetic disease of the sarcomere that causes otherwise unexplained cardiac hypertrophy and is associated with sudden death. While previous studies showed the role of the epigenetic modifier Brg1 in mouse models of HCM, additional work is needed to identify its role in humans. We tested the hypothesis that BRG1 expression is increased in periods of cardiac remodeling during fetal growth and in development of HCM. We employed immunohistochemical staining to evaluate protein expression of BRG1 in 796 human cardiac specimens (81 from patients with HCM) and describe elevated BRG1 expression in human fetal hearts in early development. In addition, we not only demonstrate increased expression of BRG1 in HCM, but we also show that other diseases that lead to heart failure have similar BRG1 expression to healthy controls. Inhibition of BRG1 in human induced pluripotent stem cell-derived cardiomyocytes significantly decreases *MYH7* and increases *MYH6*, suggesting a regulatory role for BRG1 in the pathological imbalance of the two myosin heavy chain isoforms in human HCM. These data are the first demonstration of BRG1 as a specific biomarker for human HCM and provide foundation for future studies of epigenetics in human cardiac disease.

## Introduction

Hypertrophic cardiomyopathy (HCM) is a common, heritable form of heart disease that leads to sudden death in people of all ages^1^. A variety of mutations for HCM have been identified, most commonly involving genes which encode the sarcomeric apparatus^2^; however, studies have reported varied positivity rates of molecular genetic testing among those clinically diagnosed with HCM^3–5^. Investigation of aberrant epigenetic biomarkers, those that alter a gene’s expression without altering the gene itself, remains sparse in HCM^6^. Landmark animal studies demonstrated Brg1, a critical subunit of the chromatin modifying SWI/SNF complex, is activated during normal cardiac development, repressed in the adult mouse heart, and reactivated in the setting of HCM due to transcriptional reprogramming^7,8^, and Hang and colleagues showed in a small sample of 4 HCM patients that BRG1 was elevated^8^. We evaluated nearly 800 total patient samples, 81 of which carried a clinical diagnosis of HCM using BRG1 immunohistochemistry analysis. We determined that BRG1 expression is increased in HCM hearts relative to both healthy control hearts and those with other forms of cardiac disease, which may support the use of BRG1 as a novel biomarker of HCM in the clinical setting. Further, we examined 20 human fetal cardiac specimens and concluded that BRG1 expression is increased in early gestation and repressed in later gestation, suggesting BRG1 may play an important role in human heart development. This developmental role of BRG1 was further studied using human induced-pluripotent stem cell-derived cardiomyocytes (hiPSC-CMs) in vitro to show that BRG1 inhibition prevents the normal maturation transcriptional program in immature cardiomyocytes by changing the transcriptional balance of the alpha and beta myosin heavy chain transcripts, *MYH6* and *MYH7*. These data lay the foundation for a possible mechanistic role for BRG1 in both normal cardiac development and HCM and support the idea that BRG1 could be used clinically as a sensitive biomarker for HCM.

Diagnosis of hypertrophic cardiomyopathy often requires a multidisciplinary approach, incorporating clinical, radiological, and pathological findings, as the differential diagnosis for HCM can be varied and challenging^9^. From a histopathologic perspective, it is typically associated with findings such as myocyte hypertrophy, myocyte disarray, interstitial fibrosis, and abnormal intramural vasculature^10–12^. Given the rather non-specific nature of the histopathologic findings in HCM, and the lack of a hitherto specific biomarker for a tissue diagnosis, cardiac biopsy for HCM is a Class IIb recommendation, and generally not performed^13^. To identify mutations in patients with a diagnosis of HCM and inform necessary care for relatives, clinical genetic testing is conducted. However, the mutations in HCM are widely varied^3,4^, and identification of a biomarker of HCM could provide diagnostic utility as well as possible mechanistic insight into the initiation of pathologic hypertrophy.

The finding in a landmark mouse study that Brg1 is implicated in the fetal gene program is consistent with prevailing understanding of hypertrophic cardiomyopathy, in which a hallmark of disease is the reversion of the adult heart to a fetal transcriptional program. In humans, this is characterized by an isotype switch from the adult β-myosin heavy chain (coded for by *MYH7*) to the fetal ⍺-myosin heavy chain (coded for by *MYH6*)^14^. Thus, we hypothesized that the human BRG1 is active in human fetal cardiac development and repressed in adulthood to favor the adult cardiac transcriptional program. In addition, we hypothesize that BRG1 is subsequently re-expressed in hypertrophic cardiomyopathy, where it then acts to reactivate the fetal transcriptional program to cause hypertrophic disease. Thus, BRG1 expression may demonstrate clinical utility as a biomarker of disease.

**Figure 1:**
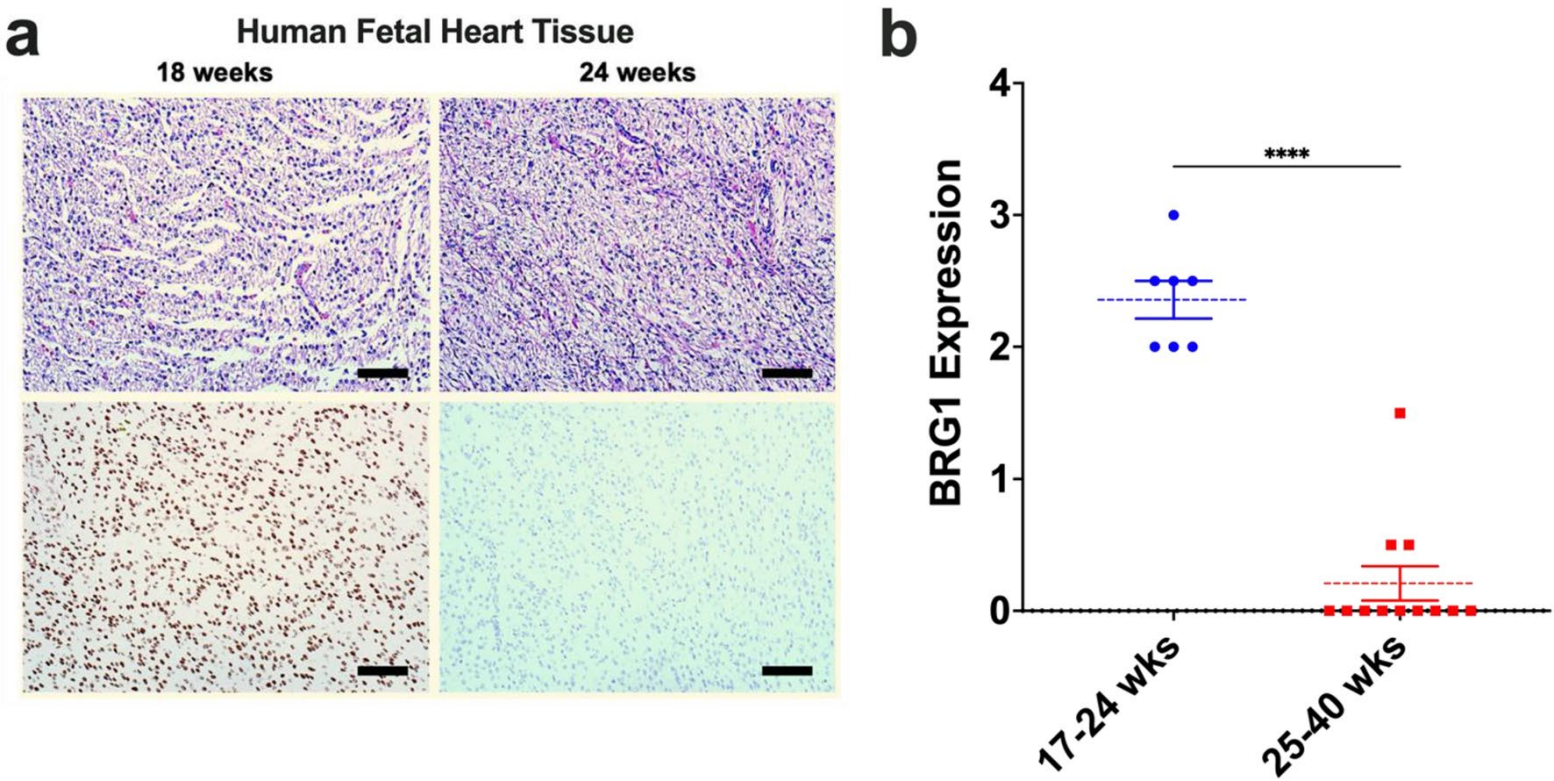
Fetal cardiac tissue expresses BRG1 prior to 24 weeks’ gestation. (**a**) SMARCA4/BRG1 expression visualized at 18 weeks and 24 weeks. Top panel shows H&E stained sample, bottom panel shows high power BRG1 immunohistochemistry. Scale bar represents 100 µm. (**b**) BRG1 expression is significantly elevated in fetal heart tissue in the first 17–24 weeks of development. Fetal cardiac tissue beyond 25 weeks’ gestations shows no significant difference in BRG1 expression relative to healthy adult controls. Data show proportions of subjects that fell into each BRG1 expression tier. ** denotes p < 0.01. Graph generated with GraphPad Prism 9.1.1 (GraphPad, San Diego, CA) by JCS.

To test these hypotheses, we employed BRG1 immunohistochemical (IHC) staining to evaluate protein expression in 796 human cardiac specimens. IHC staining of 20 fetal cardiac tissue from fetuses aged 17–40 weeks for BRG1 protein was performed. Protein expression was semi-quantitatively scored blindly by a single, board-certified cardiac pathologist (0–1 negative expression, 1–1.5 weak positive, 1.5–2.5 moderate positive, 2.5–3.0 strong positive). BRG1 protein expression was diffusely strong in developing fetal hearts (mean 2.5) up to age 24 weeks (Fig. 1a). Non-linear fit of the protein expression of BRG1 in fetal tissue (R^2^ = 0.77) suggested a significant decrease in expression at approximately 25 weeks’ gestation, a trend that was confirmed by grouping fetal tissue to pre-and post-25-week groups (Fig. 1b). Analysis of gestationally aged tissues demonstrated a near complete loss of expression at 25 weeks that was sustained throughout non-diseased adulthood.

**Figure 2:**
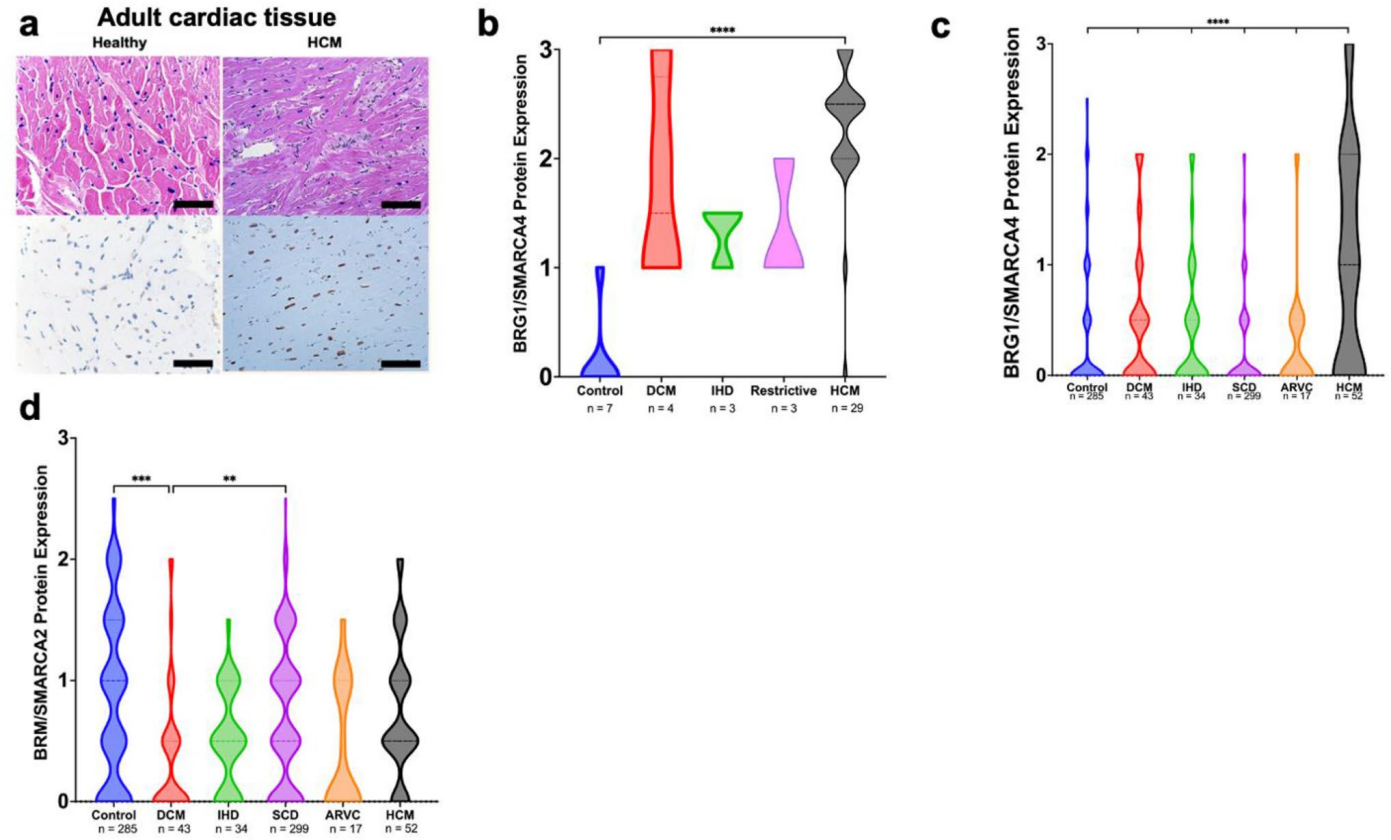
Adult cardiomyocytes express elevated protein levels of BRG1/SMARCA4 in hypertrophic cardiomyopathy. (**a**) BRG1/SMARCA4 protein expression visualized in healthy adult cardiomyocytes (left) and adult cardiomyocytes with HCM (right). Top panel shows H&E stained sample, bottom panel shows high power BRG1/SMARCA4 immunohistochemistry. Scale bars represent 50 µm. (**b**) HCM tissue in discovery cohort show increased BRG1/SMARCA4 protein expression relative to healthy controls. Data presented as mean + SEM. **** denotes p < 0.0001. (**c**) HCM tissue in validation cohort show increased BRG1/SMARCA4 protein expression relative to healthy controls. Data show proportions of subjects that fell into each BRG1/SMARCA4 protein expression tier. (**d**) HCM tissue in validation cohort do not show a significant difference in BRM/SMARCA2 protein expression compared to controls. However, DCM hearts show reduced BRM/SMARCA2 protein expression relative to healthy controls, IHD and SCD hearts. Data presented as mean + SEM. *** denotes p < 0.001, ** denotes p < 0.01. Graphs generated with GraphPad Prism 9.1.1 (GraphPad, San Diego, CA) by JCS.

IHC for BRG1 in adult cardiac specimens was subsequently performed in a small discovery cohort of 46 patients from a single institution. The cohort (median age 57; 42.3% female) included 39 diseased cardiac specimens [29 HCM, 4 dilated cardiomyopathy (DCM), 3 ischemic heart disease (IHD), 3 restrictive cardiomyopathy] from patients who underwent cardiac surgery at Duke University Hospital between 2008–2019 and 7 normal adult hearts procured at autopsy. HCM tissue was obtained from clinically diagnosed patients who underwent surgical myectomy or heart transplantation. In this discovery cohort, nuclear BRG1/SMARCA4 protein expression was significantly increased in HCM, but not non-HCM groups, relative to control hearts (p < 0.0001) (Figs. 2a,b). There was no significant effect of sex or age on BRG1/SMARCA4 protein expression (p = 0.74 by unpaired *t* test and p = 0.28, respectively). Protein expression of BRM/SMARCA2, the only other ATPase in the SWI/SNF complex, was not altered in HCM hearts, but was significantly reduced in DCM relative to controls (Fig. 2d).

To validate the findings from the initial discovery cohort, a larger cohort of 730 patients was used. This validation cohort (median age 52; 31.4% female) consisted of tissue microarray (TMA) cores of heart tissues taken from 730 patients [17 arrhythmogenic cardiomyopathy (ARVC), 43 DCM, 52 HCM, 34 IHD, 299 sudden cardiac death (SCD) and 285 control hearts] at three institutions as described previously^15^. In this larger validation cohort, BRG1/SMARCA4 protein expression was significantly greater in HCM tissues compared to other cardiac disease and control groups (p < 0.0001) (Fig. 2c). Again, there was no significant effect of sex or age (p = 0.89 by unpaired *t* test and p = 0.29, respectively). Interestingly, no significant differences in BRG1/SMARCA4 protein expression existed between patients that harbored HCM-causing mutations (n = 11), and those without genetic testing (n = 63, p = 0.33). As a biomarker of clinically diagnosed HCM in this study population, BRG1/SMARCA4 protein expression had a sensitivity of 91.7% and a specificity of 61.9%.

**Figure 3:**
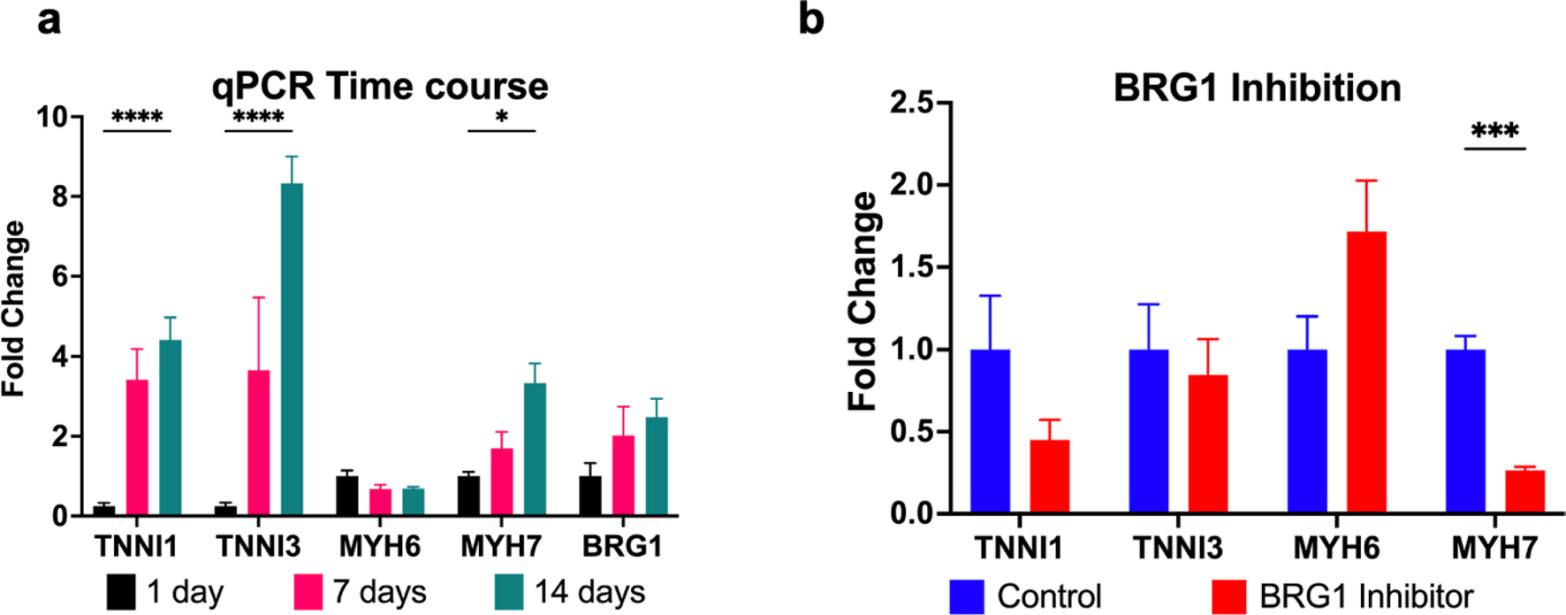
BRG1 inhibition reduces gene expression of MYH7 without altering cardiac troponin. (**a**) Quantification of *TNNI1, TNNI3, MYH6, MYH7*, and *BRG1* expression during the first 2 weeks following cardiac differentiation from human iPSCs demonstrates increased *MYH7* and *TNNI3*, as well as elevated *BRG1*. (**b**) Inhibition of BRG1 significantly reduces *MYH7* (p < 0.05). Data presented as log_2_ fold change ± SEM. Graph generated with GraphPad Prism 9.1.1 (GraphPad, San Diego, CA) by JCS.

Using hiPSC-CMs, we analyzed mRNA expression of two well-characterized pairs of cardiomyocyte protein isoforms, cardiac troponin I (*TNNI3)* and slow skeletal troponin I (*TNNI1)*, and ⍺- and β-myosin heavy chain to quantify typical transcriptional changes of maturing hiPSC-CMs in vitro. During the first 2 weeks post-cardiac differentiation, *MYH7* and *TNNI3* significantly increased (Fig. 3a), consistent with the cardiomyocyte maturation process^16^. Intriguingly, inhibition of BRG1 with the small molecule PFI-3 resulted in a significant decrease in *MYH7* expression in hiPSC-CMs and a concomitant, non-significant increase in *MYH6* (Fig. 3b). This may suggest a direct regulatory role for BRG1 in controlling the balance of *MYH6* and *MYH7* translation during early human cardiac development. However, more robust investigation is needed to understand how BRG1 exerts this control.

In this study we demonstrate that BRG1 is highly expressed in the fetal state during cardiac development, downregulated during fetal growth, and notably elevated in HCM relative to other heart diseases. These data are consistent with prior studies that showed a direct correlation of Brg1 expression in fetal cardiac tissue and HCM tissue in mice^17^, illustrating that BRG1 is correlated with cardiac remodeling in humans as well. The upregulation of BRG1 during early cardiac development in human fetuses adds another dimension to our understanding of the genetics that underpin human cardiac embryology. Previous studies have implicated Brg1 in regulating the microenvironment responsible for endocardial cushion development in mice and zebrafish^18^, and the data in this study substantiate the potential involvement of BRG1 in human fetal cardiac development. Further, our pilot investigation in hiPSC-CMs suggests that BRG1 inhibition by a small molecule inhibitor significantly reduces *MYH7* expression and increases *MYH6* expression. Interestingly, this alteration in *MYH6/MYH7* expression was accompanied by a significant increase in *NPPB*, which codes for brain natriuretic peptide (data not shown). This might suggest BRG1 plays a role not only in the balance of myosin heavy chain isoforms but may regulate hypertrophy more generally. While the findings of an in vitro experiment with hiPSC-CMs must be extrapolated cautiously, it could offer insight into a regulatory role that BRG1 may play in controlling this transcriptional balance in the development of the HCM transcriptional phenotype, characterized by a pathological increased in MYH6. Further study of the interaction of BRG1 with *MYH7* expression specifically and the transcriptome more globally may provide promising therapeutic insights into the aberrant expression of MYH6 and MYH7 in the development of HCM.

One of the strengths of this study is its large patient cohort. The number of tissue specimens from distinct patients allows for identifying the utility of BRG1 as a biomarker of HCM in the setting of a cohort with differing comorbidities, level of activity, and BMI, to name a few. Our results also indicate that BRG1 is not a biomarker of heart failure in general, nor is it a biomarker for all types of ventricular hypertrophy. Indeed, BRG1 expression was significantly greater in HCM than in DCM, and significantly elevated in HCM relative to other causes of ventricular hypertrophy. This may suggest that the divergent anatomic changes seen in these disease processes could be mediated by distinct epigenetic and genetic processes. The utility of our large cohort is borne out in our own data. In the discovery cohort, only 4 DCM specimens were present, one of which had a score of 3 for BRG1/SMARCA4 expression. This had a significant effect on the subsequent statistical analysis, which initially suggested from our discovery cohort data that BRG1 was elevated in both DCM and HCM. In the much larger validation cohort, 43 DCM specimens provided a clearer picture of typical BRG1 expression in these tissues. We were thus able to conclude that HCM tissue expression of BRG1 was significantly greater than in DCM, and that DCM was not significantly different from controls in BRG1 expression. Our large patient cohort thus provides the significant advantage of being robust to the inherent variability of human clinical data.

While we provide a new understanding of the role of BRG1 in human HCM, our study is not without its limitations. Tissue samples are obtained from a small number of medical centers, thus providing limited geographical representation that may distinctly impact epigenetics. Moreover, without genetic information for all HCM patients, tissues were studied from both genetically confirmed and clinically diagnosed HCM patients (Table 1). Interestingly, there were 5 patients with a clinical diagnosis of HCM who underwent genetic testing and were found to have pathogenic mutations in non-sarcomeric HCM genes, specifically *LAMP2, LAMA4, FAH,* and *TTN.* These subjects were not included in the HCM cohort for the sake of integrity of data interpretation, but it does raise the question of how many patients with a clinical diagnosis of HCM in fact have a heritable, non-HCM cardiac disease. This invites the exciting opportunity for future studies where more robust genetic testing of clinically diagnosed HCM patients would be performed. Further, the small number of HCM patients in our cohort with a negative gene testing panel (n = 3) or mutations of unknown significance (n = 2) limit our ability to make inferences about the effect of negative genetic testing on BRG1 expression in HCM.

**Table 1:** Individual HCM-causing gene mutations in clinically diagnosed HCM subjects in the combined discovery and validation cohorts. Parenthetical annotations indicate a pathogenic (P), likely pathogenic (LP), non-pathogenic (NP) variant, or a variant of unknown significance (US).

Mutations identified in clinically diagnosed HCM patients across discovery and validation cohorts		
Mutated gene	Mutation	BRG1/SMARCA4 protein expression
*TNNT2*	285–287 GGA deletion (US)	0
*TNNT2*	GAA c-32-13T>G; c832C>T (p.Arg278Cys) (US)	2
*TNNT2*	p.R94H mutation (P)	2
*TNNT2*	GAA c-32-13T>G; c832C>T (p.Arg278Cys) (US)	3
*MYBPC3*	Trp890Ter mutation (P)	1.5
*MYBPC3*	p.Glu542Gln (E542Q), c. 1624 G>C (LP)	2.5
*MYBPC3*	c.3490 + 1 G>T, IVS31 + 1 G>T (LP)	2.5
*MYH7*	Lys 1459 Asn, Exon 32 and Val 1323 Ile, Exon 29 (US)	1
*MYH7*	p.Asn717Asp (P)	1
*MYH7*	p. Arg 453 Cys, Exon 14 (P)	2
*MYH7*	c. 2285 A>G (p. Lys 762 Arg), Exon 20 (P)	3

Increased BRG1 expression in humans during periods of cardiac remodeling is an exciting discovery with potentially significant implications. Numerous questions remain, including understanding why the upregulation of BRG1 during fetal cardiac development is associated with physiologic remodeling, while upregulation in the adult is associated with disease. A reasonable hypothesis is that BRG1 may play an integral role in important metabolic, genetic, and structural changes inherent to the cardiomyocyte maturation process. Once the cardiomyocytes are already mature, reactivation of BRG1-involved pathways may instead lead to the disease phenotypes described above. The increased use of precision medicine and genetics research tools offer promising opportunities to study these and other questions to more completely understand the role of BRG1 in cardiac remodeling.

This study provides evidence for a sensitive, novel biomarker expressed in clinically diagnosed HCM hearts that may ultimately be used for diagnostic purposes. This work lays the foundation for more complete understanding of the role of SWI/SNF complex in controlling pathologic cardiac remodeling.

## Methods

The data described in this study are available from the corresponding author upon reasonable request. All studies and experimental protocols were approved by the Duke Health Institutional Review Board. All methods were carried out in accordance with relevant guidelines and regulations. Informed consent was obtained from all subjects and/or their legal guardians or relevant legal proxies.

The discovery cohort (median age 57; 42.3% female) included 39 diseased cardiac specimens [29 HCM, 4 dilated cardiomyopathy (DCM), 3 ischemic heart disease (IHD), 3 restrictive cardiomyopathy] from patients who underwent cardiac surgery at Duke University Hospital between 2008–2019, 7 normal adult hearts procured at autopsy, and 20 normal fetal hearts. HCM tissue was obtained from clinically diagnosed patients who underwent surgical myectomy or heart transplantation.

A validation cohort (median age 52; 31.4% female) consisted of tissue microarray (TMA) cores of heart tissues taken from 730 patients [17 arrhythmogenic cardiomyopathy (ARVC), 43 DCM, 52 HCM, 34 IHD, 299 sudden cardiac death (SCD) and 285 control hearts] at three institutions as described previously^15^. All cases of sudden cardiac death were grossly and microscopically evaluated for diseases such as arrhythmogenic cardiomyopathy and hypertrophic cardiomyopathy. All cases of SCD in this cohort were negative for these entities.

### Immunohistochemistry

Immunohistochemistry (IHC) for BRG1/SMARCA4 and BRM/SMARCA2 was performed using clinical validation protocols for monoclonal anti-BRG1/SMARCA4 and anti-BRM/SMARCA2 antibodies (Santa Cruz, #sc-17796; Invitrogen #PA5-34597) (5). BRG1/SMARCA4 protein expression was scored blindly by a single cardiac pathologist (0–1 negative expression, 1–1.5 weak positive, 1.5–2.5 moderate positive, 2.5–3.0 strong positive).

### Cell culture

BJ fibroblasts from a healthy male newborn (ATCC cell line, CRL-2522) were reprogrammed episomally into hiPSCs at the Duke University iPSC Core Facility and named DU11 (Duke University clone #11), as previously described^19^. iPSCs were maintained in mTeSR media plus mTeSR Supplement (Stem Cell Technologies). As previously described^19,20^, cardiac differentiation was induced with Activin A and ChIR 99021 followed by IWR-1 in RPMI basal media plus B27 Minus Insulin (ThermoFisher). Metabolic selection with lactate-containing media occurred over 48 h, and cells were thereafter maintained in RPMI basal media plus B27 supplement. Experiments were conducted using three different batches of differentiated hiPSC-CMs to account for the variability of the differentiation process. Purity of hiPSC-CMs was assessed by FACS analysis of cardiac troponin T staining. Only differentiation batches with purity > 80% were used in this study. The BRG1 inhibitor PFI-3 (Cayman Chemical) was applied 48 h following the transition to insulin-containing media. Drug was applied for 48 h and removed 24 h prior to RNA isolation.

### Reverse-transcription quantitative polymerase chain reaction

RNA was isolated from hiPSC-CMs using RNEasy MicroKit (Qiagen). cDNA was synthesized with iScript cDNA synthesis kit (BioRad). RT-qPCR was performed using iScript Reverse Transcription kit (BioRad). Relative expression was determined using the double delta CT method. HPRT was used as a housekeeping gene. Primers used in these studies are as follows: *HPRT*: Forward: 5′-TGACACTGGCAAAACAATGCA-3′, Reverse: 5′-GGTCCTTTTCACCAGCAAGCT-3′. *MYH6*: Forward: 5′-ACCAACCTGTCCAAGTTCCG-3′, Reverse: 5′-TTGCTTGGCACCAATGTCAC-3′, *MYH7*: Forward: 5′-CACAGCCATGGGAGATTCGG-3′, Reverse: 5′-CAGGCACGAAGACATCCTTCT-3′, *TNNI1*: 5′-ATGCCCGGAAGTCGAGAGAAAA-3′, Reverse: 5′-TCGTATCGCTCCTCATCCAC-3′, *TNNI3*: Forward: 5′-CCTCACTGACCCTCCAAACG-3′, Reverse: 5′-GAGGTTCCCTAGCCGCATC-3′.

### Statistical analysis

Statistical analysis of IHC staining was performed using Kruskal–Wallis test followed by Dunn’s multiple comparisons, or, where only two independent groups were compared, an unpaired *t* test with Welch’s correction. Significance level was set to *p* < 0.05; all results utilize Kruskal–Wallis unless otherwise stated.

Statistical analysis of RT-qPCR data was performed using one-way ANOVA with Sidak’s multiple comparisons test. Statistical analysis was conducted using GraphPad Prism 9.1.1 (GraphPad, San Diego, CA) and was used to generate the figures.

## Data Availability

Raw data will be made available upon reasonable request to the corresponding author. The RT-qPCR and genotype data generated and analyzed in this study is publicly available on Mendeley Data through the following permanent link: https://data.mendeley.com/datasets/9ktgfbvy3j/1.
